# Total Endovascular Aortic Repair in a Jehovah’s Witness Due to Chronic Postdissection Aortic Aneurysm with Endovascular Aortic Septotomy with Electrosurgery

**DOI:** 10.1093/icvts/ivaf254

**Published:** 2025-10-24

**Authors:** Georg Hagleitner, Peter Benedikt, Peter Pichler, Andreas Zierer

**Affiliations:** Central Radiology Institute, Kepler University Hospital, Linz 4020, Austria; Department of Virtual Morphology, Johannes Kepler University Linz, MED Campus I, Linz 4020, Austria; Department of Cardiothoracic and Vascular Surgery, Johannes Kepler University Linz, Kepler University Hospital, Linz 4020, Austria; Central Radiology Institute, Kepler University Hospital, Linz 4020, Austria; Department of Cardiothoracic and Vascular Surgery, Johannes Kepler University Linz, Kepler University Hospital, Linz 4020, Austria

**Keywords:** endovascular aortic repair, chronic post-dissection aortic aneurysm, patient blood management, endovascular aortic septotomy with electrosurgery

## Abstract

A 63-year-old male Jehovah’s Witness with a history of Stanford type B aortic dissection presented after a stable course over the years with a rapid expansion of the thoraco-abdominal aorta, necessitating intervention. Due to religious beliefs prohibiting blood transfusions, open surgical treatment of the aortic arch was not viable. Instead, endovascular aortic septotomy with electrosurgery of the abdominal aorta and endovascular repair of the aortic arch were successfully performed, followed by thoracic endovascular aortic repair to ensure sufficient lumen expansion for further prosthetic deployment. Five months later, the total endovascular repair of the aorta was completed by fenestrated endovascular aortic repair. Two type II endoleaks in the thoracic and abdominal aorta were treated with a percutaneous embolization. The patient remained in good general condition at the follow-up examinations, with no neurological abnormalities.

The case highlights the feasibility and advantages of established and emerging endovascular techniques as alternative to open aortic surgery for patients who refuse blood transfusions.

## INTRODUCTION

Open surgical treatment of aortic pathologies involving the aortic arch is highly invasive, requiring cardiopulmonary bypass and blood transfusions. These procedures carry a high and difficult-to-calculate risk for Jehovah’s Witnesses. For such patients, total endovascular repair offers a feasible alternative, reducing operative morbidity and eliminating the need for blood products.^1^

This case is notable for 2 reasons: First, the successful total endovascular repair from the aortic arch down to the iliac arteries in a JW patient, avoiding open surgery, and second, the use of endovascular aortic septotomy with electrosurgery (EASE) to facilitate optimal prosthesis deployment.^2^ This underscores the potential of endovascular techniques in complex aortic repairs.

## CASE PRESENTATION

A 63-year-old male JW with a history of Stanford type B aortic dissection in 2008 was under regular surveillance with computed tomography (CT) to monitor possible aneurysmal degeneration. Relevant vascular risk factors included arterial hypertension and chronic kidney disease.

**Figure 1.**
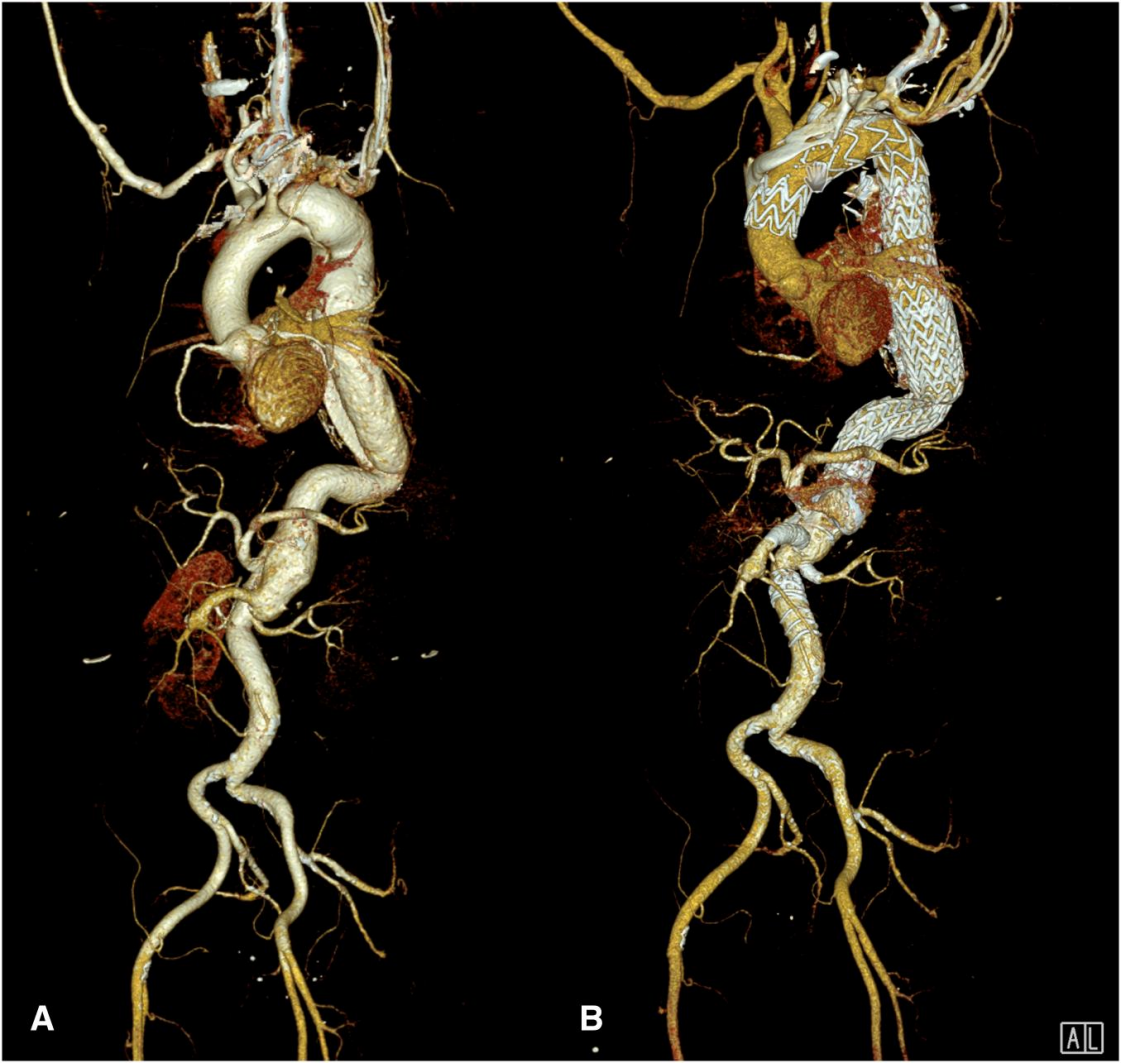
CT 3 D Volume Rendering Before Treatment (A) and After Treatment (B).

**Figure 2.**
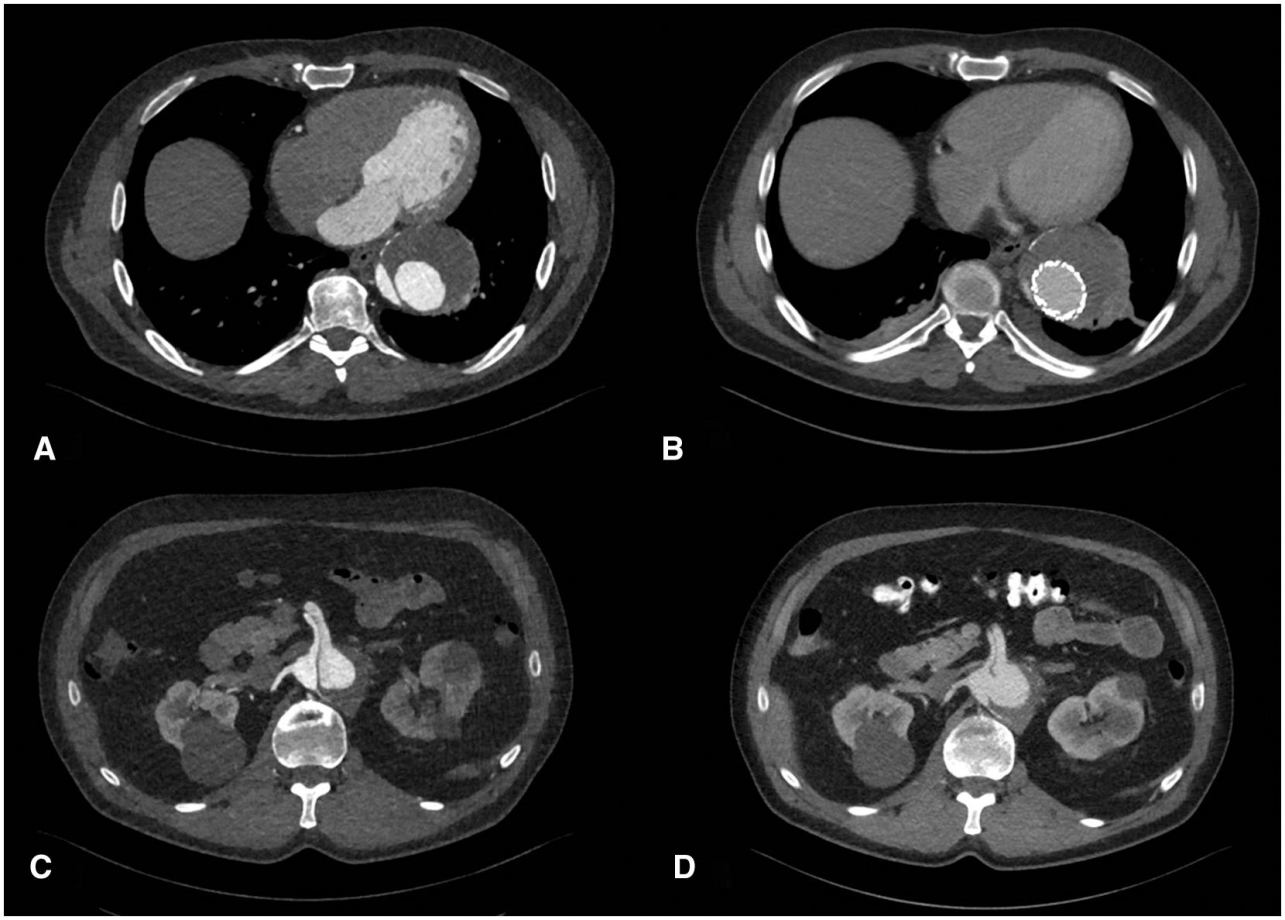
Axial Contrast-Enhanced CT Images before (2A, C) and after (2 B, D) EASE and TEVAR. Thoracic Aortic Diameter up to 6.8 × 7.5 cm; Abdominal Aortic Diameter up to 5.4 × 4.9 cm.

Between 2018 and 2023, CT scans documented a continuous size increase in the aorta. In the mid-descending aorta, dimensions grew from 5.0 × 5.6 cm to 6.8 × 7.5 cm. In the abdominal aorta, they increased from 3.7 × 4.5 cm to 4.9 × 5.4 cm (Figures 1A and 2A and 2C). Given the anatomical extent, a hybrid approach combining open surgical repair with the frozen elephant trunk technique, and endovascular repair with EASE and fenestrated endovascular aortic repair (FEVAR) was initially preferred. Due to religious beliefs, the patient declined blood transfusions; thus multidisciplinary team decision for total endovascular repair was made (Video S1).

### First endovascular procedure

Due to variable perfusion of visceral and renal vessels from the true and false lumen and true lumen compression, EASE was necessary before FEVAR. An Astato 20 0.014″ wire (Asahi Intecc Japan) was denuded with a scalpel and folded in a V-shape at about 100 cm from the proximal wire end. The prepared wire was snared from the true to the false lumen via a re-entry tear at the level of the coeliac trunk and was established as a through-and-through wire. Two protective balloons were loaded and inflated from both wire ends. After connecting the proximal wire end to an electrosurgery pencil and placing the V-shape over the dissection membrane, the EASE procedure was performed under fluoroscopy, applying electrosurgical bursts of 80 W in cutting mode.

Subsequently, a custom-made, 2-fold fenestrated aortic arch endoprosthesis with an inner branch for the left subclavian artery was successfully implanted to address the pathology in the distal aortic arch (Zone 3) and then extended with thoracic endovascular aortic repair (TEVAR) to the level of the coeliac trunk. The common lumen created by the EASE procedure enabled good perfusion of the visceral and renal arteries. (Figure 1B and D) The patient was discharged on postoperative day 6.

### Second endovascular procedure

Five months later, a custom-made 4-fold FEVAR (Anaconda, Terumo Aortic) was implanted to treat the abdominal aortic aneurysm, allowing time for the development of collateral circulation to support spinal cord perfusion. The patient was discharged on postoperative day 3.

### Third endovascular procedure and percutaneous embolization

A follow-up CT scan revealed 2 type II endoleaks: one in the thoracic aorta and the othe one in the abdominal aorta. An endovascular approach to embolize the endoleaks was unsuccessful; therefore, a percutaneous cone-beam CT-guided direct sac puncture of the thoracic and abdominal endoleak was performed, resulting in successful embolization. (Figure 1B)^3^ The patient was discharged on postoperative day three.

All 3 interventions were uneventful, with no bleeding complications or spinal cord ischaemia.

### Follow-up

At the 18-month follow-up CT-scan, the endoprostheses were appropriately positioned, with no evidence of endoleaks. Since the last examination 6 months ago, the aneurysm size has significantly reduced.

## DISCUSSION

Open surgical repair remains the gold standard for complex aortic arch pathologies providing superior long-term stabilization. However, open surgery is associated with a high risk of bleeding and a potential need for blood products, making it an unsuitable option for JW patients.^4^^,^^5^ Total endovascular repair is an alternative for high-risk surgical candidates. Advances in graft technology and implantation techniques have improved outcomes, with FEVAR offering the best long-term results in elective cases. In urgent situations, off-the-shelf branched or physician-modified grafts offer a viable alternative. However, due to high rates of reintervention after FEVAR, further standardization and careful long-term observation are necessary for its remote outcomes. The supplementary EASE procedure facilitates the creation of a common lumen by uniting the true and false lumens, ensuring sufficient space for stent graft deployment. The adaptability of endovascular techniques to complex anatomical conditions highlights the need for further research to standardize EASE, particularly concerning indications, safety, efficacy, and long-term outcomes.

## CONCLUSION

Total endovascular aortic repair combined with EASE offers a safe and effective alternative to open surgery for complex aortic pathologies in patients who cannot accept blood transfusions. This approach enabled successful treatment of extensive aortic disease with low morbidity and preserved spinal cord function. Further studies are needed to standardize these techniques and evaluate their long-term outcomes.

## Supplementary Material

ivaf254_Supplementary_Data

## Data Availability

The data that support the findings of this study are available from the corresponding author, Peter Benedikt, upon reasonable request. Some data may not be made available because of privacy or ethical restrictions.
