# Supplementary material for: Total Endovascular Aortic Repair in a Jehovah’s Witness Due to Chronic Postdissection Aortic Aneurysm with Endovascular Aortic Septotomy with Electrosurgery
Source: Interdiscip Cardiovasc Thorac Surg. 2025 Oct 24;40(11):ivaf254. doi: 10.1093/icvts/ivaf254 (PMC12596465; doi:10.1093/icvts/ivaf254)
Supplement: ivaf254_Supplementary_Data [file ivaf254_supplementary_data.zip › Supplementary Materials - ICVTS-2025-100139R3.docx]

**Supplementary Materials**

Video 1: Detailed documentation of the entire procedure and investigations

Title: Total endovascular aortic repair in a Jehovah's Witness due to chronic postdissection aortic aneurysm with endovascular aortic septotomy with electrosurgery
